# A new monoclinic polymorph of *N*-(3-methyl­phen­yl)eth­oxy­carbo­thio­amide: crystal structure and Hirshfeld surface analysis

**DOI:** 10.1107/S2056989017016280

**Published:** 2017-11-17

**Authors:** Mukesh M. Jotani, Chien Ing Yeo, Edward R. T. Tiekink

**Affiliations:** a Department of Physics, Bhavan’s Sheth R. A. College of Science, Ahmedabad, Gujarat 380 001, India; b Research Centre for Crystalline Materials, School of Science and Technology, Sunway University, 47500 Bandar Sunway, Selangor Darul Ehsan, Malaysia

**Keywords:** crystal structure, carbo­thio­amide, hydrogen bonding, Hirshfeld surface analysis

## Abstract

The title compound, C_10_H_13_NOS, is a second monoclinic polymorph (space group *P*2_1_/*c*, *Z*′ = 2) of the previously reported *C*2/*c* (*Z* = 1) polymorph [Tadbuppa & Tiekink (2005 ▸). *Z. Kristallogr. New Cryst. Struct*. **220**, 395–396]. Two independent mol­ecules comprise the asymmetric unit of the new polymorph and each of these exists as a thioamide–thione tautomer. In each molecule, the central CNOS chromophore is strictly planar [r.m.s. deviations = 0.0003 and 0.0015 Å] and forms dihedral angles of 6.17 (5) and 20.78 (5)° with the N-bound 3-tolyl rings, thereby representing the major difference between the mol­ecules. The thione-S and thio­amide-N—H atoms are *syn* in each mol­ecule and this facilitates the formation of an eight-membered thio­amide {⋯SCNH}_2_ synthon between them; the dimeric aggregates are consolidated by pairwise 3-tolyl-C—H⋯S inter­actions. In the extended structure, supra­molecular layers parallel to (102) are formed *via* a combination of 3-tolyl-C—H⋯π(3-tol­yl) and weak π–π inter­actions [inter-centroid distance between 3-tolyl rings = 3.8535 (12) Å]. An analysis of the Hirshfeld surfaces calculated for both polymorphs reveals the near equivalence of one of the independent mol­ecules of the *P*2_1_/*c* form to that in the *C*2/*c* form.

## Abstract 2

Two mol­ecules comprise the asymmetric unit in the title thio­amide mol­ecule, each of which exists as the thioamide–thione tautomer. In the crystal, the mol­ecules assemble *via* an eight-membered thio­amide {⋯SCNH}_2_ synthon to form dimeric aggregates.

## Chemical context   

Mol­ecules of the general formula *R*OC(=S)N(H)*R*′ [*R* = alkyl, ar­yl], *O*-thiocarbamates, are readily prepared from the reaction of an alcohol, *R*OH, with an iso­thio­cyanide derivative, *R*′N=C=S. Since the first report of the structure of EtOC(=S)N(H)Ph (Taylor & Tiekink, 1994 ▸), these mol­ecules have attracted the inter­est of the crystal engineering community. This inter­est arises primarily because of the propensity of these mol­ecules to form thio­amide-N—H⋯S(thione) hydrogen bonds (Ho *et al.*, 2005 ▸; Kuan *et al.*, 2007 ▸; Slater *et al.*, 2016 ▸) and the ability of these mol­ecules to form co-crystals with pyridyl-like mol­ecules (Ellis *et al.*, 2009 ▸). The neutral mol­ecules can complex bis­(phosphane)copper(I) chloride to reveal fascinating intra­molecular phenyl-C—H⋯π(quasi-chelate ring) inter­actions where the π-system is the hydrogen-bond mediated (CuCl⋯HNCS) ring (Yeo *et al.*, 2014 ▸); inter­molecular versions of C—H⋯π(quasi-chelate ring) inter­actions are also known (Zukerman-Schpector *et al.*, 2016 ▸). The anions form very stable compounds with phosphanegold(I) moieties to yield luminescent materials in the solid state (Ho *et al.*, 2006 ▸) as well as potential anti-bacterial (Yeo *et al.*, 2013 ▸) and anti-cancer (Ooi *et al.*, 2017 ▸) agents. It was in the latter context that the title polymorph (I) was discovered. Thus, (I) was synthesized afresh for complexation to phosphanegold(I) and during characterization exhibited distinctive crystallographic properties from a previously described material, *i.e*. a *C*2/*c* form (Tadbuppa & Tiekink, 2005 ▸), hereafter (Ic). In the present report, the crystal and mol­ecule structures of a new monoclinic polymorph of (I), *i.e*. (Ip), are described along with a Hirshfeld surface analysis of both polymorphs, conducted in order to discover distinctive packing patterns.
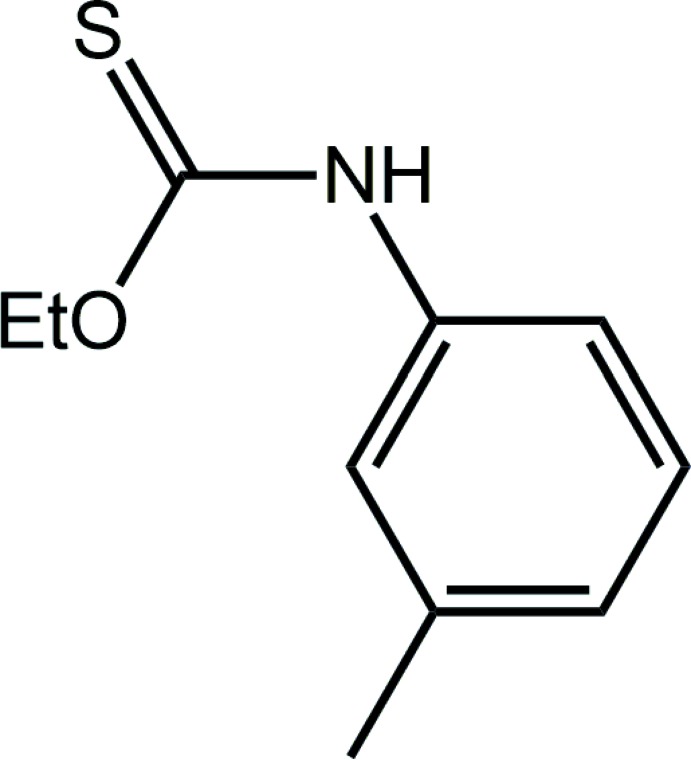


## Structural commentary   

**Figure 1:**
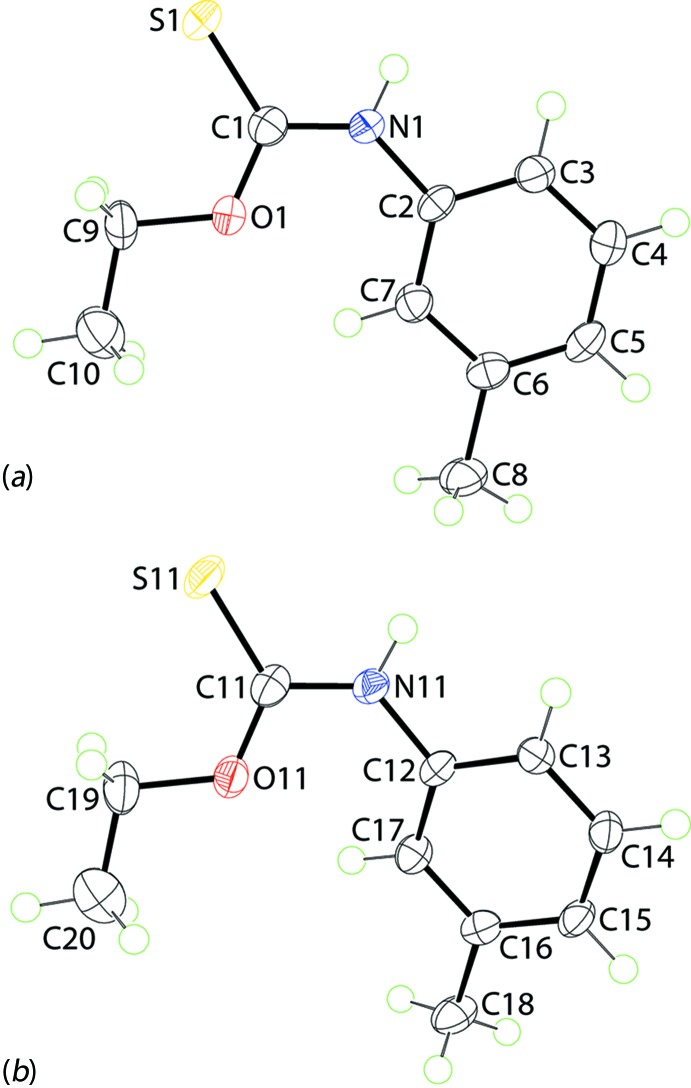
The mol­ecular structures of the two independent mol­ecules comprising the asymmetric unit of (Ip) showing the atom-labelling scheme and displacement ellipsoids at the 70% probability level.

**Figure 2:**
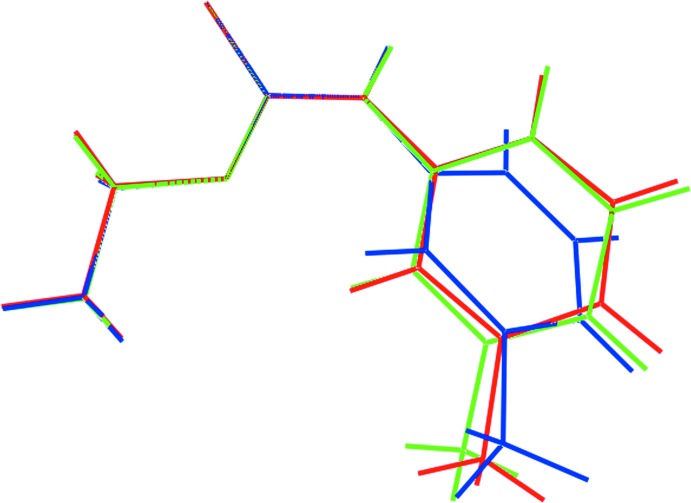
Overlay diagram of the two independent mol­ecules of (Ip) (S1-mol­ecule, red image; S11-mol­ecule, green) and that of the original *C*2/*c* polymorph (blue image), (Ic). The mol­ecules have been superimposed so that the central S, O and N atoms are coincident.

The crystallographic asymmetric unit of (Ip), Fig. 1 ▸, comprises two independent mol­ecules which are chemically indistinguishable, Table 1 ▸. The thione-S and thio­amide-N—H atoms are *syn* in each mol­ecule and each exists as a thio­amide–thione tautomer. The central OC(=S)N chromophores are strictly planar with the r.m.s. deviation of the four fitted atoms being 0.0003 Å [0.0015 Å for the S11-mol­ecule]. The bond lengths follow the expected trends with the C1—O1, N1 bonds being significantly shorter than the C9—O1 and C2—N1 bonds, respectively. The angles about the quaternary atom vary systematically, with those involving the thione-S1 atom being greater than the O1—C1—N1 bond angle. Of the bond angles involving the thione-S1 atom, the angle involving the O1 atom is greater by 2–3° than that formed by the sterically less encumbered N1 atom. The major difference between the key geometric parameters listed in Table 1 ▸ is found in the angles subtended at the N1 atom with the angle for the S1-mol­ecule being nearly 3° wider than that for the S1-mol­ecule. There is also a conformational difference between the two mol­ecules, readily qu­anti­fied in terms of the dihedral angles formed between the central chromophore and 3-tolyl rings of 6.17 (5) and 20.78 (5)° for the S1- and S11-mol­ecules, respectively. As seen from the overlay diagram, Fig. 2 ▸, the ethyl groups have an open conformation and overlap closely with the C1—O1—C9—C10 and C11—O11—C19—C20 torsion angles being −178.76 (17) and 177.42 (18)°, respectively.

Geometric parameters for the original polymorph of (I), *i.e*. (Ic), are also included in Table 1 ▸. A comparison of these show the values in (Ip) and (Ic) to be equal within experimental error and those of (Ic) often lying between the two independent values found for (Ip). As evidenced from Fig. 2 ▸, there is a greater twist in the mol­ecule as indicated by the dihedral angle of 30.44 (6)° formed between the central chromophore and the 3-tolyl ring. The orientation of the O-bound ethyl group is as for both mol­ecules of (Ip) with the C1—O1—C9—C10 torsion angle being −176.96 (17)°.

## Supra­molecular features   

**Figure 3:**
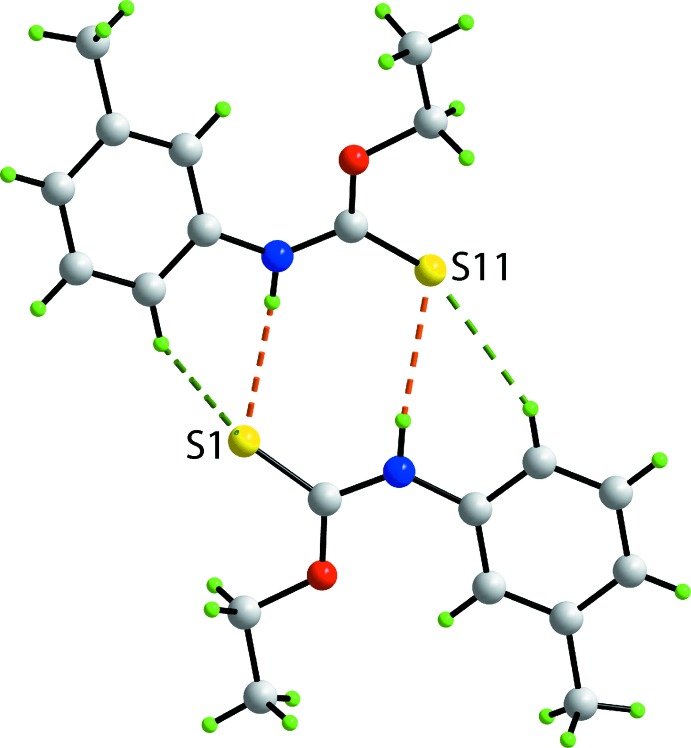
A view of the supra­molecular dimer in (Ip) sustained by thio­amide-N—H⋯S(thione) hydrogen bonds and supported by 3-tolyl-C—H⋯S(thione) inter­actions, shown as orange and green dashed lines, respectively.

**Figure 4:**
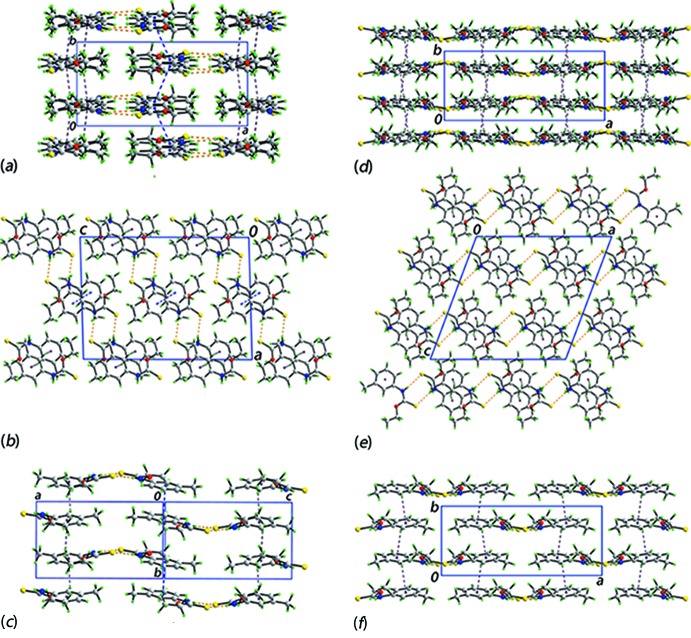
Mol­ecular packing in (Ip): (*a*) a view of the unit-cell contents shown in projection down the *c* axis, (*b*) a view of the unit-cell contents shown in projection down the *b* axis and (*c*) a view of the supra­molecular layer. Mol­ecular packing in (Ic): (*d*) a view of the unit-cell contents shown in projection down the *c* axis, (*e*) a view of the unit-cell contents shown in projection down the *b* axis and (*f*) a view of the supra­molecular layer. The thioamide-N—H⋯S(thione), C—H⋯π and π–π inter­actions are shown as orange, purple and blue dashed lines, respectively.

The most notable feature of the mol­ecular packing of (I) is the presence of an eight-membered thio­amide synthon, {⋯SCNH}_2_, formed *via* thioamide-N—H⋯S(thione) hydrogen bonds, between the two independent mol­ecules comprising the asymmetric unit, Fig. 3 ▸ and Table 2 ▸. As shown in Fig. 3 ▸, the N—H⋯S hydrogen bonds are supported by 3-tolyl-C—H⋯S inter­actions, Table 2 ▸, with that involving the S1 atom being slightly beyond the standard distance criteria in *PLATON* (Spek, 2009 ▸). Globally, like mol­ecules stack along the *b*-axis direction. The S1-mol­ecules are connected *via* weak π–π inter­actions between the 3-tolyl rings with the inter-centroid distance being 3.8535 (12) Å for the symmetry operation 1 − *x*, 2 − *y*, 1 − *z*. The connections between the S11-mol­ecules are of the type 3-tolyl-C—H⋯π(3-tol­yl), Table 2 ▸. The columns pack into alternating layers of S1- and S11-mol­ecules parallel to [001], Fig. 4 ▸ *a*, and connections between them are made through the thioamide-N—H⋯S(thione) hydrogen bonds mentioned above, resulting in supra­molecular layers parallel to (102), Fig. 4 ▸ *b*. The layers, Fig. 4 ▸ *c*, stack with no directional inter­actions between them.

The mol­ecular packing in (Ic) has not been discussed in any detail (Tadbuppa & Tiekink, 2005 ▸) and hence, is now described. The eight-membered thio­amide synthon, {⋯SCNH}_2_, seen in the packing of (Ip) is also found in the packing of (Ic), Table 3 ▸, with an important difference, that being the synthon has crystallographic twofold symmetry; the putative 3-tolyl-C—H⋯S inter­action is long at 2.92 Å. Globally, mol­ecules pack into columns parallel to the *b* axis and are sustained by 3-tolyl-C—H⋯π(3-tol­yl) inter­actions, Fig. 4 ▸ *d* and Table 3 ▸. Connections between columns are made by the aforementioned thioamide-N—H⋯S(thione) hydrogen bonds. The result is supra­molecular layers that stack along the *c* axis, Fig. 4 ▸ *e*. A view of the layer is shown in Fig. 4 ▸ *f*.

From the images of Fig. 4 ▸, it is obvious that despite some similarities, the mol­ecular packing in polymorphs (Ip) and (Ic) are distinct. This point is highlighted in the analysis of the Hirshfeld surfaces of (Ip) and (Ic) discussed in the next section.

## Analysis of the Hirshfeld surfaces of (Ip) and (Ic)   

The Hirshfeld surfaces for the individual mol­ecules in (Ip), overall (Ip) and for (Ic) were calculated in accord with a recent report on a pair of polymorphs (Kuan *et al.*, 2017 ▸). The calculations clearly reveal the similarities and differences in the inter­molecular inter­actions instrumental in the crystals of the polymorphs.

**Figure 5:**
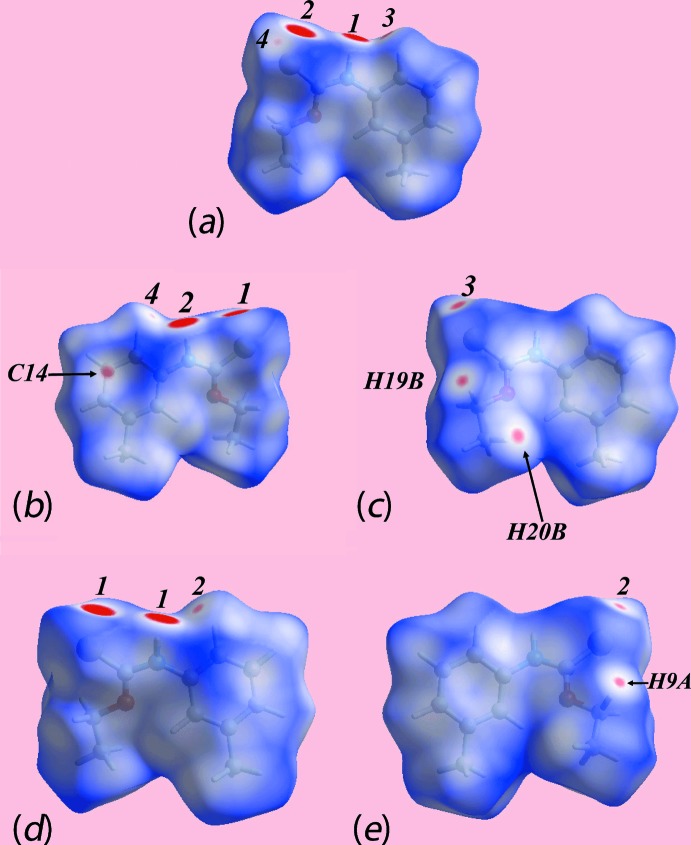
Views of the Hirshfeld surfaces mapped over *d*_norm_ for the (*a*) S1-containing mol­ecule of (Ip) in the range −0.147 to +1.345 au, (*b*) and (*c*) S11-containing mol­ecule in (Ip) in the range −0.149 to +1.274 au and (*d*) and (*e*) mol­ecule of polymorph (Ic) in the range −0.109 to 1.397 au.

**Figure 6:**
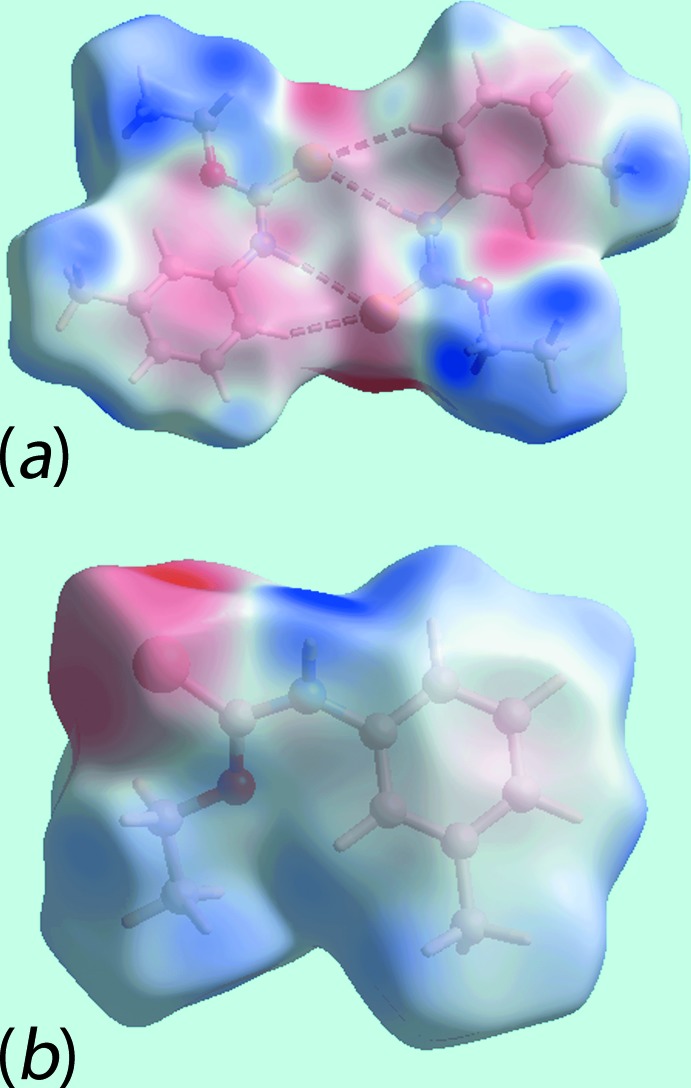
Views of Hirshfeld surfaces mapped over the electrostatic potential for (*a*) the asymmetric unit of (Ip) in the ±0.046 au range and (*b*) mol­ecule of (Ic) in ±0.069 au range. The red and blue regions represent negative and positive electrostatic potentials, respectively.

**Figure 7:**
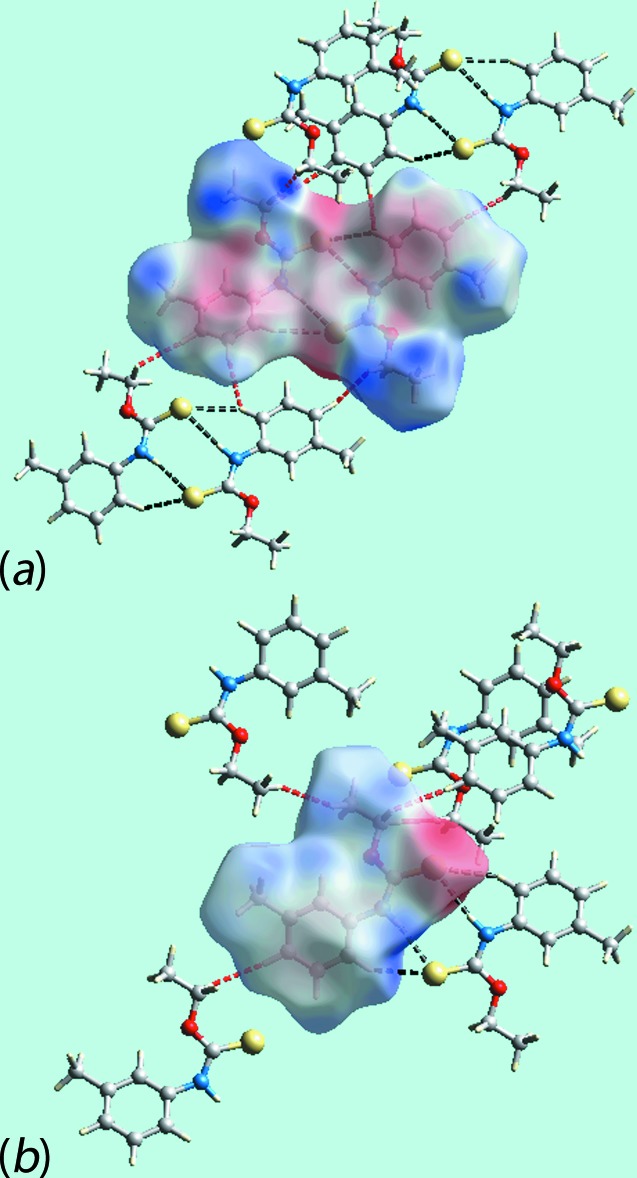
Views of the Hirshfeld surfaces about a reference mol­ecule mapped over the electrostatic potential highlighting the short inter­atomic H⋯H contacts (red dashed lines) and inter­molecular N—H⋯S and C—H⋯ S inter­actions (black dashed lines) in (*a*) (Ip) and (*b*) (Ic).

The appearance of bright-red spots near the thioamide-H and thione-S atoms, diminutive red spots near the 3-tolyl-H, eth­oxy-H atoms and thione-S atoms on the Hirshfeld surfaces mapped over *d*_norm_ shown in Fig. 5 ▸ for both independent mol­ecules of (Ip) as well as for polymorph (Ic) are indicative of comparable thioamide-N—H⋯S(thione) and 3-tolyl-C—H⋯S(thione) inter­actions, and short inter­atomic H⋯H contacts in their respective crystals, Table 4 ▸; values in Table 4 ▸ were obtained from an analysis employing the *CrystalExplorer* package (Wolff *et al.* 2012 ▸). As there are two independent mol­ecules in monoclinic polymorph (Ip), it exhibits a pair of the above-mentioned inter­molecular inter­actions shown with labels 1 to 4 in Fig. 5 ▸ *a*–*c*, whereas in form (Ic) they are labelled as 1 and 2 in Fig. 5 ▸ *d* and *e*. In addition to the above, the faint-red spots viewed near 3-tolyl-C14 in Fig. 5 ▸ *b* and eth­oxy-H20*B* in Fig. 5 ▸ *c* indicate the significance of short inter­atomic C⋯H/H⋯C contacts, Table 4 ▸, in the packing of (Ip). The donors and acceptors of inter­molecular inter­actions are also represented with blue and red regions, respectively, on the Hirshfeld surfaces mapped over electrostatic potential in Fig. 6 ▸. The new monoclinic polymorph (Ip) has distinct and a greater number of short inter­atomic contacts than for (Ic) owing, in part, to the presence of two distinct mol­ecules per asymmetric unit, Table 4 ▸. The short inter­atomic H⋯H contacts together with inter­molecular N—H⋯S and C—H⋯S inter­actions formed with the atoms of reference mol­ecules within Hirshfeld surfaces mapped over electrostatic potential for (Ip) and (Ic) are highlighted in Fig. 7 ▸.

**Figure 8:**
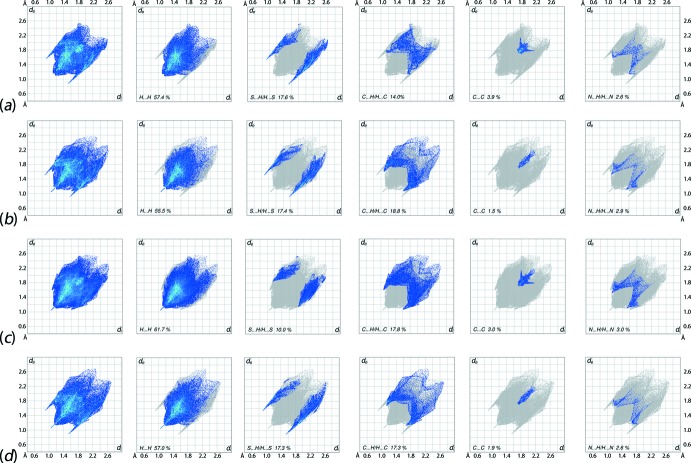
The full two-dimensional fingerprint plot and those delineated into H⋯H, S⋯H/H⋯S, C⋯H/H⋯C, C⋯C and N⋯H/H⋯N (left to right) contacts for (*a*) S1-mol­ecule of (Ip), (*b*) S11-mol­ecule of (Ip), (*c*) overall (Ip) and (*d*) (Ic).

The overall two-dimensional fingerprint plots for the S1 and S11-containing mol­ecules of (Ip), the whole asymmetric unit of (Ip) and for the polymorph (Ic) are illustrated in Fig. 8 ▸ *a*–*d*, respectively. In addition, the fingerprint plots delineated into H⋯H, S⋯H/H⋯S, C⋯H/H⋯C, C⋯C and N⋯H/H⋯N contacts (McKinnon *et al.*, 2007 ▸) are included in Fig. 8 ▸; the relative contributions from different inter­atomic contacts to the Hirshfeld surfaces are summarized in Table 5 ▸. The nearly similar distribution of points in the fingerprint plots for S11-containing mol­ecule of (Ip) and that of (Ic) indicate similarity in their mol­ecular environments although some of the equivalent inter­atomic distances differ, Tables 2 ▸–4 ▸ ▸.

The fingerprint plots delineated into H⋯H contacts, Fig. 8 ▸ *b* and *d*, have needle-like tips pointing at *d*_e_ + *d*_i_ ∼ 2.1 Å indicating short inter­atomic H⋯H contacts, Table 4 ▸, for the S11-containing mol­ecule of (Ip) and for (Ic), both involving eth­oxy-H atoms. The other short inter­atomic contacts in both forms are characterized from the points located within the pair of short peaks in (Ip) and a single short peak in (Ic), respectively, at *d*_e_ + *d*_i_ < 2.4 Å, *i.e*. at the sum of their van der Waals radii.

The involvement of eth­oxy-H atoms in short inter­atomic C⋯H/H⋯C contacts decreases the percentage contribution from H⋯H contacts to the Hirshfeld surface of the S11-containing mol­ecule whereas the contribution from equivalent contacts to the surface of the S1-containing mol­ecule of (Ip) and that of (Ic) are almost same, Table 5 ▸. The increase in percentage contribution from these contacts to the Hirshfeld surface of overall asymmetric unit of (Ip) is due to the inter­molecular N—H⋯S and C—H⋯S inter­actions between the respective atoms of S1- and S11-containing mol­ecules thereby decreasing the contribution from S⋯H/H⋯S contacts to the overall surface, Table 5 ▸. This fact is confirmed from the nearly same percentage contribution from S⋯H/H⋯S contacts to the Hirshfeld surfaces of the individual S1- and S11-containing mol­ecules of (Ip) and of the mol­ecule of the (Ic) form, Table 5 ▸, and also from pair of forceps-like tips at *d*_e_ + *d*_i_ ∼ 2.6 Å with the nearly same distribution of points in their respective fingerprint plots in Fig. 8 ▸.

The similar distribution of points in the fingerprint plot delineated into C⋯H/H⋯C contacts for the S11-containing mol­ecule of (Ip), Fig. 8 ▸ *b*, and of (Ic), Fig. 8 ▸ *d*, indicate their involvement in the inter­molecular C—H⋯π contacts showing pairs of tips at *d*_e_ + *d*_i_ ∼ 2.8 and 2.9 Å, respectively. This is confirmed by the slight increase in the percentage contribution from these contacts to the Hirshfeld surface of the S11-containing mol­ecule of (Ip) *cf*. the S1-containing mol­ecule, Table 5 ▸. In other words, the contribution from C⋯H/H⋯C contacts to the surface of the S1-containing mol­ecule of (Ip), Table 5 ▸, is decreased due to the absence of C—H⋯π contacts involving this mol­ecule whereas the greater percentage contribution from C⋯C contacts to the Hirshfeld surface of this mol­ecule results from the presence of π–π stacking inter­actions between the symmetry-related 3-tolyl rings. This is also evident from the arrow-like distribution of points around *d*_e_ = *d*_i_ = 1.8 Å in the C⋯C delineated fingerprint plot shown in Fig. 8 ▸ *a*.

The contribution of 3.0% from N⋯H/H⋯N contacts to the Hirshfeld surface of whole asymmetric unit of polymorph (Ip) indicate the presence of short inter­atomic N⋯H/H⋯N contacts between the thioamide-N1 and tolyl-H18*B* atoms, Table 4 ▸, although all of the delineated fingerprint plots have a similar distributions of points, Fig. 8 ▸, at least to a first approximation. The other inter­atomic contacts summarized in Table 4 ▸ make only small contributions to the Hirshfeld surfaces and have negligible contributions on the respective mol­ecular packings.

## Database survey   

**Figure 9:**
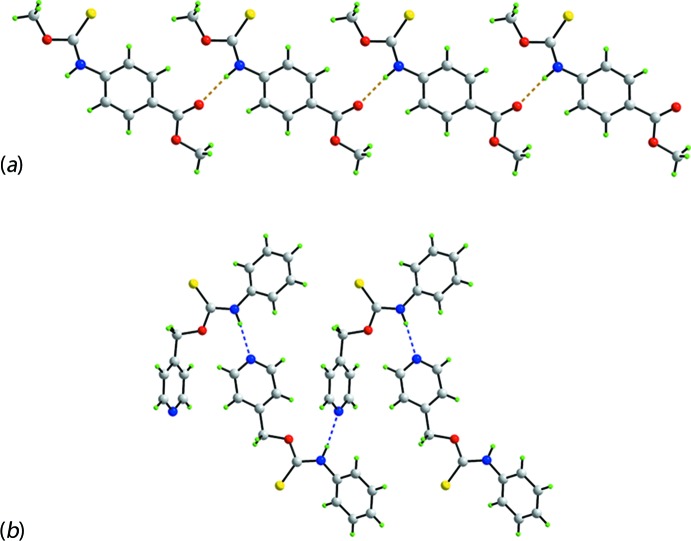
Supra­molecular aggregation in related carbo­thio­amide structures: (*a*) linear supra­molecular chain in the crystal of MeOC(=S)N(H)(4-C(=O)Me-phen­yl) (VI) mediated by thio­amide-N—H⋯O(carb­oxy) hydrogen bonding shown as orange dashed lines and (*b*) zigzag chain in the crystal of (4-pyrid­yl)CH_2_OC(=S)N(H)phenyl mediated by thio­amide-N—H⋯N(pyrid­yl) hydrogen bonding shown as blue dashed lines.

According to a search of the Cambridge Structural Database (Version 5.38, May update; Groom *et al.*, 2016 ▸), there are 22 monofunctional carbo­thio­amide mol­ecules related to the title compound, with (Ip) and (Ic) being the only pair of polymorphs characterized thus far. Referring to Table 6 ▸, the overwhelming majority of the 24 crystallographically characterized thio­amides feature an eight-membered thio­amide, {⋯SCNH}_2_, synthon. Thus, in 13 of the characterized structures, the synthon is formed about a centre of inversion, motif **A**. In five structures, two independent mol­ecules (*Z*′ = 2) comprise the asymmetric unit, as in (Ip), and these associated *via* the {⋯SCNH}_2_ synthon but with no crystallographically imposed symmetry, motif **A′**. There is a single example of a structure with *Z*′ = 3 (Taylor & Tiekink, 1994 ▸). Here, one of the independent mol­ecules self-associates about a centre of inversion, as in motif **A**, whereas the two remaining independent mol­ecules are connected by the {⋯SCNH}_2_ synthon, as found in motif **A′**. This is motif **A′′**. Two structures feature the {⋯SCNH}_2_ synthon located about a twofold axis of symmetry, as in (Ic), *i.e*. motif **A′′′**. The remaining three structures do not feature thioamide-N—H⋯S(thione) hydrogen bonding. In the structure of MeOC(=S)N(H)(4-C(=O)Me-phen­yl) (VI) (Ho *et al.*, 2005 ▸), motif **B**, thioamide-N—H⋯O(carb­oxy) hydrogen bonding is observed, leading to a linear supra­molecular chain as shown in Fig. 9 ▸ *a*. This structure is noteworthy as being the only example where the conformation of the thio­amide moiety is *anti* rather than the normally observed *syn*. The final variation, motif **C**, is found in two structures, Table 6 ▸. The structure of (4-pyrid­yl)CH_2_OC(=S)N(H)phenyl (XIX) (Xiao *et al.*, 2006 ▸) serves as an exemplar. Thus, in the crystal of (XIX), thioamide-N—H⋯N(pyrid­yl) hydrogen bonds lead to a zigzag chain as shown in Fig. 9 ▸ *b*. In summary, an inspection of the data in Table 6 ▸ indicates the predominance of thioamide-N—H⋯S(thione) hydrogen bonding in these carbo­thio­amides, at least in the absence of competing synthons, as seen in motifs **B** and **C** 
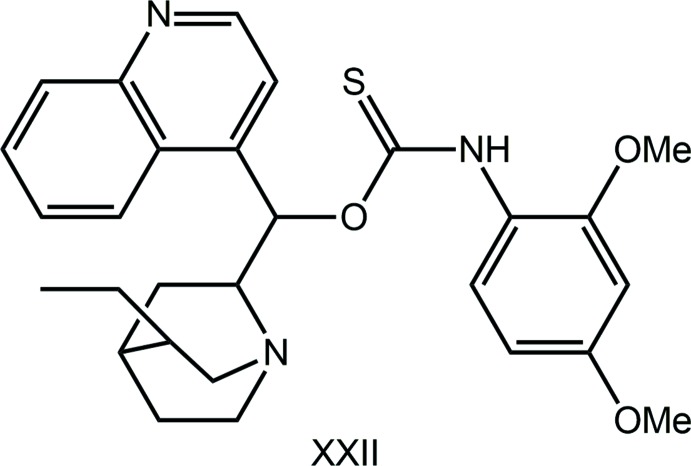
.

## Synthesis and crystallization   

All chemicals and solvents were used as purchased without purification. To prepare (Ip), 3-tolyl iso­thio­cyanate (Merck; 2.5 mmol, 0.34 ml) was added to NaOH (Merck; 2.5 mmol, 0.10 g) in EtOH (Merck; 3 ml) and the mixture was stirred at room temperature for 2 h, followed by the addition of excess 5 *M* HCl solution. The resulting mixture was stirred for another 1.5 h. The final product was extracted with chloro­form (Merck; 10 ml) and left for evaporation at room temperature, yielding brown crystals after 1 week. M.p. (Krüss KSP1N melting point meter): 339–340 K. IR (Perkin Elmer Spectrum 400 FT Mid-IR/Far-IR spectrophotometer; cm^−1^): 3211 (*s*) (N—H), 1451 (*s*) (C—N), 1209 (*s*) (C=S), 1064 (*s*) (C—O).

## Refinement   

Crystal data, data collection and structure refinement details are summarized in Table 7 ▸. The carbon-bound H atoms were placed in calculated positions (C—H = 0.95–0.99 Å) and were included in the refinement in the riding-model approximation, with *U*_iso_(H) set to 1.2–1.5*U*_eq_(C). The nitro­gen-bound H atoms were located in a difference Fourier map but were refined with a distance restraint of N—H = 0.88±0.01 Å, and with *U*_iso_(H) set to 1.2*U*_eq_(N). Owing to poor agreement, one reflection, *i.e*. (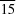 1 4), was omitted from the final cycles of refinement.

## Supplementary Material

Crystal structure: contains datablock(s) I, global. DOI: 10.1107/S2056989017016280/hb7715sup1.cif

Structure factors: contains datablock(s) I. DOI: 10.1107/S2056989017016280/hb7715Isup2.hkl

CCDC reference: 1585129

Additional supporting information:  crystallographic information; 3D view; checkCIF report

## Figures and Tables

**Table 1:** Selected geometric parameters (Å, °) in (Ip) and (Ic)

Parameter	(Ip), S1-mol­ecule	(Ip), S11-mol­ecule^*a*^	(Ic)
C1—S1	1.6768 (19)	1.6752 (19)	1.6720 (18)
C1—O1	1.321 (2)	1.319 (2)	1.325 (2)
C1—N1	1.338 (2)	1.339 (2)	1.337 (2)
C9—O1	1.457 (2)	1.454 (2)	1.451 (2)
C2—N1	1.421 (2)	1.423 (2)	1.426 (2)
S1—C1—O1	124.23 (14)	125.00 (15)	124.53 (12)
S1—C1—N1	122.06 (14)	121.61 (15)	122.11 (13)
O1—C1—N1	113.71 (16)	113.39 (16)	113.37 (15)
C1—O1—C9	118.72 (15)	119.01 (15)	119.29 (15)
C1—N1—C2	132.48 (16)	129.60 (16)	130.17 (15)

Note: (*a*) add 10 to atom labels to tally with the numbering in Fig. 1 ▸ *b*.

**Table 2:** Hydrogen-bond geometry (Å, °) *Cg*1 is the centroid of the (C12–C17) ring.

*D*—H⋯*A*	*D*—H	H⋯*A*	*D*⋯*A*	*D*—H⋯*A*
N1—H1*N*⋯S11	0.87 (1)	2.62 (1)	3.4859 (16)	174 (2)
N11—H11*N*⋯S1	0.87 (1)	2.54 (1)	3.3985 (15)	171 (2)
C3—H3⋯S11	0.95	2.86	3.708 (2)	150
C13—H13⋯S1	0.95	2.94	3.7090 (19)	139
C17—H17⋯*Cg*1^i^	0.95	2.82	3.471 (2)	127

Symmetry code: (i) 
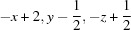
.

**Table 3:** Hydrogen-bond geometry (Å, °) for (Ic) *Cg*1 is the centroid of the (C2–C7) ring.

D—H⋯A	D—H	H⋯A	D⋯A	D—H⋯A
N1—H1n⋯S1^i^	0.87	2.58	3.4142 (16)	160
C7—H7⋯*Cg*1^ii^	0.94	2.91	3.4973 (17)	122

Symmetry code: (i) −*x*, *y*, 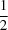 − *z*; (ii) 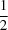 − *x*, −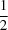 + *y*, 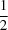 − *z*.

**Table 4:** Summary of short inter­atomic contacts (Å) in (Ip) and (Ic)^*a*^

Contact	Distance	Symmetry operation
(Ip)		
H3⋯H14	2.35	*x*, 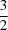 − *y*, − 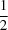 + *z*
H5⋯H9*B*	2.28	*x*, 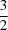 − *y*, 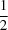 + *z*
H15⋯H19*A*	2.31	*x*, 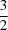 − *y*, − 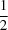 + *z*
H19*B*⋯H19*B*	2.08	2 − *x*, 2 − *y*, 1 − *z*
C10⋯H20*C*	2.87	−1 + *x*, *y*, *z*
C14⋯H20*B*	2.77	2 − *x*, − 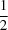 + *y*, 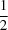 − *z*
N1⋯H18*B*	2.73	2 − *x*, − 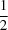 + *y*, 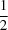 − *z*
(Ic)		
H7⋯H9*b*	2.37	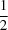 + *x*, 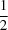 − *y*, 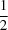 + *z*
H9*a*⋯H9*a*	2.11	−*x*, −*y*, −*z*
H10*b*⋯H10*b*	2.32	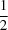 − *x*, 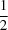 − *y*, −*z*

Note: (*a*) the atom numbering for the mol­ecule in (Ic) follows that for the S1-mol­ecule in (Ip).

**Table 5:** Percentage contributions of inter­atomic contacts to the Hirshfeld surfaces for the individual mol­ecules in (Ip), overall (Ip) and (Ic)

Contact	Percentage contribution			
	(Ip), S1-mol­ecule	(Ip), S11-mol­ecule	overall (Ip)	(Ic)
H⋯H	57.4	55.5	61.7	57.0
S⋯H/H⋯S	17.6	17.4	10.0	17.3
C⋯H/H⋯C	14.0	18.8	17.8	17.3
C⋯C	3.9	1.5	3.0	1.9
N⋯H/H⋯N	2.6	2.9	3.0	2.6
C⋯O/O⋯C	2.5	2.5	2.7	2.4
O⋯H/H⋯O	1.0	1.2	1.2	1.1
C⋯N/N⋯C	0.9	0.3	0.6	0.4

**Table 6:** Hydrogen-bonding patterns in *R*OC(=S)N(H)*R*′

Number	*R*	*R*′	*Z*′	Hydrogen bonds	Motif	Refcode	Ref.
(II)	Me	phen­yl	1	N—H⋯S	**A**	OJIHAQ	Ho *et al.* (2003 ▸)
(III)	Me	4-NO_2_-phen­yl	1	N—H⋯S	**A**	CAZFUF	Ho *et al.* (2005 ▸)
(IV)	Me	4-*C*(=O)OMe-phen­yl	1	N—H⋯S	**A**	CAZGAM	Ho *et al.* (2005 ▸)
(V)	Me	4-Cl-phen­yl	2	N—H⋯S	**A′**	CAZCEQ	Ho *et al.* (2005 ▸)
(VI)	Me	4-*C*(=O)Me-phen­yl	1	N—H⋯O	**B**	CAZGIU	Ho *et al.* (2005 ▸)
(VII)	Me	2-tol­yl	1	N—H⋯S	**A**	TAZSIX	Kuan *et al.* (2005 ▸)
(VIII)	Me	4-tol­yl	2	N—H⋯S	**A′**	TIBYUZ	Ho *et al.* (2007 ▸)
(IX)	Et	phen­yl	3	N—H⋯S	**A′′**	PINPIL	Taylor & Tiekink (1994 ▸)
(Ip)	Et	3-tol­yl	2	N—H⋯S	**A′**	–	This work
(Ip)	Et	3-tol­yl	1	N—H⋯S	**A′′′**	TAZTUK	Tadbuppa & Tiekink (2005 ▸)
(*X*)	Et	4-tol­yl	1	N—H⋯S	**A**	TIBYOT	Tadbuppa & Tiekink (2007*a* ▸)
(XI)	Et	3-OMe-phen­yl	1	N—H⋯S	**A**	UDUPAL	Hanif *et al.* (2007 ▸)
(XII)	Et	4-NO_2_-phen­yl	1	N—H⋯S	**A**	NENLAU	Benson *et al.* (2006 ▸)
(XIII)	Et	4-Cl-phen­yl	1	N—H⋯S	**A**	DEYQEE	Tadbuppa & Tiekink (2007*b* ▸)
(XIV)	*n*-Pr	phen­yl	2	N—H⋯S	**A′**	PAWKAB	Sudkaow *et al.* (2012 ▸)
(XV)	*i*-Pr	Ph	1	N—H⋯S	**A**	ADOGUW	Kuan *et al.* (2007 ▸)
(XVI)	*i*-Pr	4-tol­yl	1	N—H⋯S	**A**	ADOGOQ	Kuan *et al.* (2007 ▸)
(XVII)	*i*-Pr	4-Cl-phen­yl	1	N—H⋯S	**A**	ADOHAD	Kuan *et al.* (2007 ▸)
(XVIII)	*i*-Pr	4-NO_2_-phen­yl	1	N—H⋯S	**A**	MISDEY	Ellis *et al.* (2008 ▸)
(XIX)	4-pyridyl­meth­yl	phen­yl	2	N—H⋯N	**C**	IFACOI	Xiao *et al.* (2006 ▸)
(XX)	*i*-Bu	phen­yl	1	N—H⋯S	**A′′′**	KEQJAS	Jian *et al.* (2006 ▸)
(XXI)	2,4-Me_2_-phen­yl	phen­yl	1	N—H⋯S	**A**	POVVOL	Abraham *et al.* (1995 ▸)
(XXII)	2,4-(OMe)_2_-phen­yl	*R* ^1*a*^	1	N—H⋯N	**C**	OSIZOG	Zhou *et al.* (2010 ▸)
(XXIII)	Cy	phen­yl	2	N—H⋯S	**A′**	VEFKUO	Sahoo *et al.* (2012 ▸)

Note: (*a*) see Scheme 2 for the chemical diagram of (XXII).

**Table 7:** Experimental details

Crystal data	
Chemical formula	C_10_H_13_NOS
*M*_r_	195.27
Crystal system, space group	Monoclinic, *P*2_1_/*c*
Temperature (K)	100
*a*, *b*, *c* (Å)	14.3999 (5), 7.0388 (3), 19.9725 (7)
β (°)	91.727 (3)
*V* (Å^3^)	2023.45 (13)
*Z*	8
Radiation type	Mo *K*α
μ (mm^−1^)	0.28
Crystal size (mm)	0.20 × 0.20 × 0.05

Data collection	
Diffractometer	Agilent SuperNova, Dual, Mo at zero, Atlas
Absorption correction	Multi-scan (*CrysAlis PRO*; Agilent, 2011 ▸)
*T*_min_, *T*_max_	0.662, 1.000
No. of measured, independent and observed [*I* > 2σ(*I*)] reflections	15588, 4577, 3514
*R*_int_	0.040
(sin θ/λ)_max_ (Å^−1^)	0.651

Refinement	
*R*[*F* ^2^ > 2σ(*F* ^2^)], *wR*(*F* ^2^), *S*	0.047, 0.125, 1.03
No. of reflections	4577
No. of parameters	245
No. of restraints	2
H-atom treatment	H atoms treated by a mixture of independent and constrained refinement
Δρ_max_, Δρ_min_ (e Å^−3^)	0.72, −0.24

Computer programs: *CrysAlis PRO* (Agilent, 2011 ▸), *SHELXS97* (Sheldrick, 2008 ▸), *SHELXL2014* (Sheldrick, 2015 ▸), *ORTEP-3 for Windows* (Farrugia, 2012 ▸), *QMol* (Gans & Shalloway, 2001 ▸), *DIAMOND* (Brandenburg, 2006 ▸) and *publCIF* (Westrip, 2010 ▸).

## supplementary crystallographic information

### Crystal data

**Table:**

C_10_H_13_NOS	*F*(000) = 832
*M**_r_* = 195.27	*D*_x_ = 1.282 Mg m^−^^3^
Monoclinic, *P*2_1_/*c*	Mo *K*α radiation, λ = 0.71073 Å
*a* = 14.3999 (5) Å	Cell parameters from 6144 reflections
*b* = 7.0388 (3) Å	θ = 2.4–27.5°
*c* = 19.9725 (7) Å	µ = 0.28 mm^−^^1^
β = 91.727 (3)°	*T* = 100 K
*V* = 2023.45 (13) Å^3^	Slab, colourless
*Z* = 8	0.20 × 0.20 × 0.05 mm

### Data collection

**Table:**

Agilent SuperNova, Dual, Mo at zero, Atlas diffractometer	4577 independent reflections
Radiation source: SuperNova (Mo) X-ray Source	3514 reflections with *I* > 2σ(*I*)
Mirror monochromator	*R*_int_ = 0.040
Detector resolution: 10.4041 pixels mm^-1^	θ_max_ = 27.6°, θ_min_ = 2.5°
ω scan	*h* = −18→18
Absorption correction: multi-scan (CrysAlis PRO; Agilent, 2011)	*k* = −9→9
*T*_min_ = 0.662, *T*_max_ = 1.000	*l* = −18→25
15588 measured reflections	

### Refinement

**Table:**

Refinement on *F*^2^	2 restraints
Least-squares matrix: full	H atoms treated by a mixture of independent and constrained refinement
*R*[*F*^2^ > 2σ(*F*^2^)] = 0.047	*w* = 1/[σ^2^(*F*_o_^2^) + (0.0629*P*)^2^ + 0.8006*P*] where *P* = (*F*_o_^2^ + 2*F*_c_^2^)/3
*wR*(*F*^2^) = 0.125	(Δ/σ)_max_ = 0.001
*S* = 1.03	Δρ_max_ = 0.72 e Å^−^^3^
4577 reflections	Δρ_min_ = −0.24 e Å^−^^3^
245 parameters	

### Special details

**Table:**

Geometry. All esds (except the esd in the dihedral angle between two l.s. planes) are estimated using the full covariance matrix. The cell esds are taken into account individually in the estimation of esds in distances, angles and torsion angles; correlations between esds in cell parameters are only used when they are defined by crystal symmetry. An approximate (isotropic) treatment of cell esds is used for estimating esds involving l.s. planes.

### Fractional atomic coordinates and isotropic or equivalent isotropic displacement parameters (Å^2^)

**Table:**

	*x*	*y*	*z*	*U*_iso_*/*U*_eq_	
S1	0.63595 (3)	0.62560 (8)	0.29847 (2)	0.02560 (15)	
O1	0.48087 (9)	0.6728 (2)	0.36508 (7)	0.0205 (3)	
N1	0.61175 (11)	0.6959 (2)	0.42674 (8)	0.0176 (3)	
H1N	0.6720 (7)	0.683 (3)	0.4295 (11)	0.021*	
C1	0.57256 (13)	0.6663 (3)	0.36605 (10)	0.0183 (4)	
C2	0.57411 (12)	0.7410 (3)	0.48972 (9)	0.0157 (4)	
C3	0.63901 (13)	0.7851 (3)	0.54059 (10)	0.0198 (4)	
H3	0.7035	0.7828	0.5318	0.024*	
C4	0.60988 (14)	0.8320 (3)	0.60359 (10)	0.0224 (4)	
H4	0.6545	0.8604	0.6382	0.027*	
C5	0.51590 (14)	0.8380 (3)	0.61691 (10)	0.0214 (4)	
H5	0.4963	0.8719	0.6603	0.026*	
C6	0.45044 (13)	0.7944 (3)	0.56662 (10)	0.0189 (4)	
C7	0.47948 (13)	0.7455 (3)	0.50316 (10)	0.0185 (4)	
H7	0.4349	0.7151	0.4688	0.022*	
C8	0.34802 (13)	0.8043 (3)	0.58036 (11)	0.0258 (5)	
H8A	0.3157	0.6989	0.5576	0.039*	
H8B	0.3227	0.9252	0.5637	0.039*	
H8C	0.3391	0.7952	0.6287	0.039*	
C9	0.43042 (14)	0.6465 (3)	0.30146 (10)	0.0250 (5)	
H9A	0.4433	0.5193	0.2827	0.030*	
H9B	0.4489	0.7440	0.2687	0.030*	
C10	0.33069 (15)	0.6655 (4)	0.31611 (12)	0.0334 (5)	
H10A	0.2934	0.6493	0.2747	0.050*	
H10B	0.3192	0.7917	0.3349	0.050*	
H10C	0.3135	0.5680	0.3485	0.050*	
S11	0.85195 (3)	0.66025 (9)	0.45041 (2)	0.02577 (15)	
O11	0.99877 (9)	0.7448 (2)	0.37829 (6)	0.0192 (3)	
N11	0.86976 (10)	0.6753 (2)	0.31952 (8)	0.0175 (3)	
H11N	0.8103 (7)	0.652 (3)	0.3174 (11)	0.021*	
C11	0.91055 (13)	0.6951 (3)	0.38026 (10)	0.0185 (4)	
C12	0.90609 (12)	0.7096 (3)	0.25516 (9)	0.0156 (4)	
C13	0.84323 (12)	0.7751 (3)	0.20604 (9)	0.0179 (4)	
H13	0.7802	0.7967	0.2165	0.021*	
C14	0.87304 (13)	0.8082 (3)	0.14206 (9)	0.0193 (4)	
H14	0.8303	0.8523	0.1084	0.023*	
C15	0.96520 (13)	0.7774 (3)	0.12656 (9)	0.0192 (4)	
H15	0.9854	0.8019	0.0825	0.023*	
C16	1.02797 (13)	0.7108 (3)	0.17527 (10)	0.0177 (4)	
C17	0.99808 (13)	0.6749 (3)	0.23923 (9)	0.0176 (4)	
H17	1.0404	0.6265	0.2724	0.021*	
C18	1.12821 (13)	0.6769 (3)	0.15808 (10)	0.0245 (5)	
H18A	1.1566	0.5877	0.1904	0.037*	
H18B	1.1622	0.7975	0.1599	0.037*	
H18C	1.1308	0.6234	0.1129	0.037*	
C19	1.05091 (14)	0.7744 (3)	0.44091 (10)	0.0256 (5)	
H19A	1.0493	0.6584	0.4689	0.031*	
H19B	1.0238	0.8806	0.4663	0.031*	
C20	1.14753 (15)	0.8193 (4)	0.42346 (12)	0.0349 (5)	
H20A	1.1840	0.8490	0.4643	0.052*	
H20B	1.1477	0.9291	0.3933	0.052*	
H20C	1.1750	0.7096	0.4012	0.052*	

### Atomic displacement parameters (Å^2^)

**Table:**

	*U*^11^	*U*^22^	*U*^33^	*U*^12^	*U*^13^	*U*^23^
S1	0.0230 (3)	0.0401 (3)	0.0140 (3)	−0.0017 (2)	0.00422 (18)	−0.0020 (2)
O1	0.0190 (7)	0.0284 (8)	0.0141 (7)	0.0011 (6)	−0.0009 (5)	−0.0020 (6)
N1	0.0155 (7)	0.0236 (9)	0.0139 (8)	0.0005 (7)	0.0022 (6)	−0.0005 (7)
C1	0.0200 (9)	0.0174 (10)	0.0176 (10)	−0.0011 (8)	0.0017 (7)	0.0017 (8)
C2	0.0211 (9)	0.0129 (9)	0.0132 (9)	0.0008 (7)	0.0039 (7)	0.0011 (7)
C3	0.0182 (9)	0.0233 (11)	0.0178 (10)	0.0014 (8)	0.0019 (7)	0.0004 (8)
C4	0.0251 (10)	0.0262 (11)	0.0157 (10)	−0.0002 (9)	−0.0014 (8)	−0.0010 (8)
C5	0.0279 (10)	0.0234 (11)	0.0133 (9)	0.0026 (8)	0.0052 (7)	−0.0009 (8)
C6	0.0205 (9)	0.0181 (10)	0.0184 (10)	−0.0003 (8)	0.0052 (7)	0.0035 (8)
C7	0.0199 (9)	0.0203 (10)	0.0154 (10)	−0.0016 (8)	0.0014 (7)	0.0020 (8)
C8	0.0226 (10)	0.0331 (12)	0.0221 (11)	0.0014 (9)	0.0077 (8)	0.0024 (9)
C9	0.0254 (10)	0.0337 (12)	0.0155 (10)	−0.0020 (9)	−0.0057 (8)	−0.0002 (9)
C10	0.0267 (11)	0.0404 (14)	0.0329 (13)	−0.0041 (10)	−0.0037 (9)	−0.0003 (11)
S11	0.0225 (3)	0.0423 (3)	0.0127 (3)	0.0072 (2)	0.00474 (18)	0.0035 (2)
O11	0.0203 (7)	0.0236 (8)	0.0137 (7)	0.0012 (6)	−0.0003 (5)	−0.0007 (6)
N11	0.0147 (7)	0.0238 (9)	0.0141 (8)	0.0025 (7)	0.0027 (6)	0.0016 (7)
C11	0.0200 (9)	0.0200 (10)	0.0155 (9)	0.0075 (8)	0.0027 (7)	0.0009 (8)
C12	0.0196 (9)	0.0152 (9)	0.0121 (9)	−0.0005 (7)	0.0031 (7)	0.0003 (7)
C13	0.0154 (9)	0.0225 (10)	0.0158 (10)	−0.0017 (8)	0.0006 (7)	−0.0010 (8)
C14	0.0219 (9)	0.0217 (10)	0.0141 (9)	−0.0031 (8)	−0.0032 (7)	0.0010 (8)
C15	0.0242 (10)	0.0225 (10)	0.0110 (9)	−0.0033 (8)	0.0030 (7)	0.0001 (8)
C16	0.0198 (9)	0.0161 (9)	0.0175 (10)	−0.0014 (8)	0.0042 (7)	−0.0007 (8)
C17	0.0201 (9)	0.0165 (9)	0.0162 (10)	0.0029 (8)	0.0010 (7)	0.0010 (8)
C18	0.0236 (10)	0.0286 (12)	0.0217 (11)	0.0038 (9)	0.0090 (8)	0.0029 (9)
C19	0.0265 (10)	0.0323 (12)	0.0176 (10)	0.0042 (9)	−0.0044 (8)	−0.0055 (9)
C20	0.0302 (12)	0.0359 (14)	0.0382 (14)	−0.0012 (10)	−0.0047 (10)	−0.0008 (11)

### Geometric parameters (Å, º)

**Table:**

S1—C1	1.6768 (19)	S11—C11	1.6752 (19)
O1—C1	1.321 (2)	O11—C11	1.319 (2)
O1—C9	1.457 (2)	O11—C19	1.454 (2)
N1—C1	1.338 (2)	N11—C11	1.339 (2)
N1—C2	1.421 (2)	N11—C12	1.423 (2)
N1—H1N	0.872 (9)	N11—H11N	0.872 (9)
C2—C3	1.394 (3)	C12—C13	1.393 (3)
C2—C7	1.397 (2)	C12—C17	1.393 (2)
C3—C4	1.378 (3)	C13—C14	1.380 (3)
C3—H3	0.9500	C13—H13	0.9500
C4—C5	1.388 (3)	C14—C15	1.389 (3)
C4—H4	0.9500	C14—H14	0.9500
C5—C6	1.391 (3)	C15—C16	1.389 (3)
C5—H5	0.9500	C15—H15	0.9500
C6—C7	1.390 (3)	C16—C17	1.383 (3)
C6—C8	1.510 (3)	C16—C18	1.513 (2)
C7—H7	0.9500	C17—H17	0.9500
C8—H8A	0.9800	C18—H18A	0.9800
C8—H8B	0.9800	C18—H18B	0.9800
C8—H8C	0.9800	C18—H18C	0.9800
C9—C10	1.480 (3)	C19—C20	1.479 (3)
C9—H9A	0.9900	C19—H19A	0.9900
C9—H9B	0.9900	C19—H19B	0.9900
C10—H10A	0.9800	C20—H20A	0.9800
C10—H10B	0.9800	C20—H20B	0.9800
C10—H10C	0.9800	C20—H20C	0.9800
			
C1—O1—C9	118.72 (15)	C11—O11—C19	119.01 (15)
C1—N1—C2	132.48 (16)	C11—N11—C12	129.60 (16)
C1—N1—H1N	115.6 (15)	C11—N11—H11N	117.9 (15)
C2—N1—H1N	111.9 (15)	C12—N11—H11N	112.0 (15)
O1—C1—N1	113.71 (16)	O11—C11—N11	113.39 (16)
O1—C1—S1	124.23 (14)	O11—C11—S11	125.00 (15)
N1—C1—S1	122.06 (14)	N11—C11—S11	121.61 (15)
C3—C2—C7	119.42 (17)	C13—C12—C17	119.95 (17)
C3—C2—N1	115.45 (16)	C13—C12—N11	116.33 (16)
C7—C2—N1	125.13 (17)	C17—C12—N11	123.68 (17)
C4—C3—C2	120.17 (17)	C14—C13—C12	119.62 (17)
C4—C3—H3	119.9	C14—C13—H13	120.2
C2—C3—H3	119.9	C12—C13—H13	120.2
C3—C4—C5	120.51 (18)	C13—C14—C15	120.40 (17)
C3—C4—H4	119.7	C13—C14—H14	119.8
C5—C4—H4	119.7	C15—C14—H14	119.8
C4—C5—C6	119.90 (18)	C14—C15—C16	120.16 (17)
C4—C5—H5	120.0	C14—C15—H15	119.9
C6—C5—H5	120.0	C16—C15—H15	119.9
C7—C6—C5	119.81 (17)	C17—C16—C15	119.61 (17)
C7—C6—C8	119.97 (18)	C17—C16—C18	120.42 (18)
C5—C6—C8	120.21 (17)	C15—C16—C18	119.96 (17)
C6—C7—C2	120.18 (18)	C16—C17—C12	120.22 (18)
C6—C7—H7	119.9	C16—C17—H17	119.9
C2—C7—H7	119.9	C12—C17—H17	119.9
C6—C8—H8A	109.5	C16—C18—H18A	109.5
C6—C8—H8B	109.5	C16—C18—H18B	109.5
H8A—C8—H8B	109.5	H18A—C18—H18B	109.5
C6—C8—H8C	109.5	C16—C18—H18C	109.5
H8A—C8—H8C	109.5	H18A—C18—H18C	109.5
H8B—C8—H8C	109.5	H18B—C18—H18C	109.5
O1—C9—C10	106.11 (17)	O11—C19—C20	107.04 (17)
O1—C9—H9A	110.5	O11—C19—H19A	110.3
C10—C9—H9A	110.5	C20—C19—H19A	110.3
O1—C9—H9B	110.5	O11—C19—H19B	110.3
C10—C9—H9B	110.5	C20—C19—H19B	110.3
H9A—C9—H9B	108.7	H19A—C19—H19B	108.6
C9—C10—H10A	109.5	C19—C20—H20A	109.5
C9—C10—H10B	109.5	C19—C20—H20B	109.5
H10A—C10—H10B	109.5	H20A—C20—H20B	109.5
C9—C10—H10C	109.5	C19—C20—H20C	109.5
H10A—C10—H10C	109.5	H20A—C20—H20C	109.5
H10B—C10—H10C	109.5	H20B—C20—H20C	109.5
			
C9—O1—C1—N1	178.80 (17)	C19—O11—C11—N11	178.92 (17)
C9—O1—C1—S1	−1.1 (3)	C19—O11—C11—S11	−0.6 (3)
C2—N1—C1—O1	−3.2 (3)	C12—N11—C11—O11	−3.8 (3)
C2—N1—C1—S1	176.68 (16)	C12—N11—C11—S11	175.77 (16)
C1—N1—C2—C3	−172.1 (2)	C11—N11—C12—C13	−147.0 (2)
C1—N1—C2—C7	7.3 (3)	C11—N11—C12—C17	35.4 (3)
C7—C2—C3—C4	0.2 (3)	C17—C12—C13—C14	−1.1 (3)
N1—C2—C3—C4	179.66 (18)	N11—C12—C13—C14	−178.82 (17)
C2—C3—C4—C5	−0.7 (3)	C12—C13—C14—C15	−0.3 (3)
C3—C4—C5—C6	0.7 (3)	C13—C14—C15—C16	0.7 (3)
C4—C5—C6—C7	−0.2 (3)	C14—C15—C16—C17	0.1 (3)
C4—C5—C6—C8	−178.83 (19)	C14—C15—C16—C18	179.80 (18)
C5—C6—C7—C2	−0.3 (3)	C15—C16—C17—C12	−1.5 (3)
C8—C6—C7—C2	178.33 (18)	C18—C16—C17—C12	178.84 (18)
C3—C2—C7—C6	0.3 (3)	C13—C12—C17—C16	2.0 (3)
N1—C2—C7—C6	−179.08 (18)	N11—C12—C17—C16	179.53 (17)
C1—O1—C9—C10	−178.76 (17)	C11—O11—C19—C20	177.42 (18)

### Hydrogen-bond geometry (Å, º)

Cg1 is the centroid of the (C12–C17) ring.

**Table:**

*D*—H···*A*	*D*—H	H···*A*	*D*···*A*	*D*—H···*A*
N1—H1*N*···S11	0.87 (1)	2.62 (1)	3.4859 (16)	174 (2)
N11—H11*N*···S1	0.87 (1)	2.54 (1)	3.3985 (15)	171 (2)
C3—H3···S11	0.95	2.86	3.708 (2)	150
C13—H13···S1	0.95	2.94	3.7090 (19)	139
C17—H17···*Cg*1^i^	0.95	2.82	3.471 (2)	127

Symmetry code: (i) −*x*+2, *y*−1/2, −*z*+1/2.
